# Quality Matters: Implementation Moderates Student Outcomes in the PATHS Curriculum

**DOI:** 10.1007/s11121-017-0802-4

**Published:** 2017-06-19

**Authors:** Neil Humphrey, Alexandra Barlow, Ann Lendrum

**Affiliations:** Manchester Institute of Education, The University of Manchester, Oxford Road, Manchester, M13 9PL UK

**Keywords:** Social and emotional learning, Intervention, Implementation, Paths, Outcomes

## Abstract

Analyses of the relationship between levels of implementation and outcomes of school-based social and emotional learning (SEL) interventions are relatively infrequent and are typically narrowly focused. Thus, our objective was to assess the relationship between variability in a range of implementation dimensions and intervention outcomes in the Promoting Alternative Thinking Strategies (PATHS) curriculum. Implementation of PATHS was examined in 69 classrooms across 23 schools in the first year of a major randomized controlled trial. Implementation data were generated via classroom-level structured observations. In addition to factual data on dosage and reach, exploratory factor analysis of observer ratings revealed two distinct implementation dimensions, namely, “quality and participant responsiveness” and “procedural fidelity.” Student social-emotional skills, pro-social behavior, internalizing symptoms, and externalizing problems were captured through child self-report and teacher informant-report surveys (*N* = 1721). Hierarchical linear modeling of study data revealed that higher implementation quality and participant responsiveness was associated with significantly lower ratings of students’ externalizing problems at 12-month follow-up. Conversely, and contrary to expectations, higher dosage was associated with significantly lower pro-social behavior and social-emotional skills at 12-month follow-up. No significant associations were found between variability in either procedural fidelity or reach and any intervention outcomes. The implications of these findings are discussed, and study limitations are noted.

## Introduction

Universal, school-based social and emotional learning (SEL) interventions foster the social-emotional skills (e.g., self-management, social awareness, relationship skills) of children and young people through explicit instruction in the context of learning environments that are safe, caring, well-managed, and participatory (Weissberg et al. 2015). Three recent meta-analyses have rigorously demonstrated that SEL interventions can lead to meaningful improvements in a range of student outcomes, including their social-emotional skills, mental health, and academic attainment (Durlak et al. 2011; Sklad et al. 2012; Wigelsworth et al. 2016). However, the implementation of SEL interventions is variable, and this variability is hypothesized to be a key moderator of intervention outcomes (Durlak 2016). Our primary objective in this study, therefore, was to assess the relationship between levels of implementation and intervention outcomes in the Promoting Alternative Thinking Strategies (PATHS) curriculum. In doing so, we also sought to offer a distinct contribution to knowledge vis-à-vis the distinction between fidelity and quality in implementation and prevention science.

## Implementation of School-Based Interventions

The implementation of school-based interventions is typically conceptualized in terms of constructs such as *fidelity* (what is delivered and how closely does this adhere to intervention guidance materials?), *dosage* (how much of the intervention is delivered?), *quality* (how well is the intervention delivered?), *reach* (was the intervention delivered to all intended recipients?), and *participant responsiveness* (did recipients engage with the intervention?) (Lendrum et al. 2016). A current source of contention is whether fidelity and quality are distinct. To some, fidelity is a superordinate construct, used to describe the overall pattern of activity, with other implementation dimensions viewed as subordinate indicators (Carroll et al. 2007). This carries an implicit assumption that for intervention outcomes to be replicated, the exact delivery regime under which an intervention was validated must also be replicated. This is the “zero-sum-game” view of implementation: higher fidelity results in better outcomes, and any deviation from the intended intervention model must therefore be detrimental (e.g., Elliott and Mihalic 2004). Fidelity thus becomes synonymous with quality, to the extent that the terms are used interchangeably (Lendrum et al. 2016).

To others, *implementation* is the superordinate construct, with fidelity included as a subordinate indicator alongside the aforementioned other dimensions (Berkel et al. 2011; Durlak and DuPre 2008) and typically understood and operationalized in procedural terms (e.g., how closely the structure and sequence of activities outlined in the intervention guidance are followed). Implementation quality is viewed as distinct from fidelity, referring to how effectively the intervention has been delivered for the achievement of intended outcomes (O’Donnell 2008; Odom et al. 2010), including facets such as implementer competence and skills, enthusiasm and engagement, and preparedness for implementation (Lendrum et al. 2016). This view is adopted in the current study and is reflected in our approach to assessment (see “Method” section). We see it as more consistent with emergent theorization of implementation that acknowledges the important distinction between *what* is implemented and *how well* (Berkel et al. 2011).

Whatever model one subscribes to, there is broad agreement that measurement of implementation is still in its relative infancy: “Even if the concept of implementation is not new, the idea of developing ways of measuring it certainly is” (Ogden and Fixsen 2014, p.8). Thus, the field has yet to emerge at a clear consensus regarding the optimal frequency and timing of measurement within a period of implementation (Durlak 2015; Humphrey 2016b). However, one area where there is general agreement is modality, with independent structured observations considered to be greatly preferable to teacher self-report methods, the latter being subject to substantial positive bias (Hansen et al. 2014).

Although it is generally accepted that “implementation matters” (Durlak and DuPre 2008), the evidence base pertaining to SEL and school-based interventions more generally is currently limited in a number of respects. First, despite a significant rise in the proportion of studies reporting on implementation in the last two decades (currently up to 69% of SEL studies, Wigelsworth et al. 2016; but still less than half of school-based intervention studies more generally, Bruhn et al. 2015), most offer only descriptive data, which are used to provide evidence that a given intervention was actually delivered and thus strengthen the internal validity of trial outcome analyses. By contrast, analyses in which researchers model levels of implementation dimensions as moderators of intervention effects are relatively infrequent, despite their obvious significance in terms of both internal and external validity in program evaluation. For example, a recent systematic review found that only 10% of intervention studies reported implementation-outcome relationships (Schoenwald and Garland 2014).

Second, research published to date has been characterized by a narrow focus on particular aspects of implementation at the expense of others. Thus, while 63% of studies included in Durlak and DuPre’s (2008) seminal review assessed fidelity, only 10% assessed quality. This narrow approach reflects the zero-sum-game model noted earlier and greatly increases the risk of a Type III error (the inaccurate attribution of cause) (Lendrum et al. 2016). Less frequently studied implementation dimensions such as quality may be equally or even more important than fidelity and dosage in driving intervention outcomes (Durlak 2015). A teachers’ preparedness, ability to actively engage and enthuse students, and clarity of delivery is crucial for effective learning; without this, the internalization of lesson content and subsequent skill development that underpins intervention outcomes is unlikely to occur (Berkel et al. 2011; Lendrum et al. 2016).

Third, the conceptualization and assessment of different aspects of implementation currently lags behind other aspects of intervention research. As noted above, terms such as fidelity and quality have been used interchangeably in some studies (e.g., Social and Character Development Research Consortium 2010). Furthermore, the means by which they are measured generally lacks the level of rigor afforded to the assessment of intervention outcomes (Ogden and Fixsen 2014). A recent systematic review found that only around one third of papers provided *any* data on the psychometric properties of instruments used to generate implementation data (Schoenwald and Garland 2014). Studies reporting factor analytic work to establish the distinctiveness of implementation dimensions as the foundation for implementation-outcomes analyses are extremely rare (Cross et al. 2015; Pettigrew et al. 2015). To a certain degree, this is understandable, given that each intervention typically generates its own implementation measures (which may be used infrequently), and also that interventions often evolve over time (meaning that implementation measures would also need to be revised frequently). One possible solution is the development and application of standardized implementation measures, though existing attempts have met with mixed success to date (Humphrey et al. 2016b).

## The PATHS Curriculum

PATHS is a universal SEL intervention that aims to help all children to manage their behavior, understand their emotions, and work well with others. It is designed to be delivered by class teachers and includes a series of lessons on topics such as identifying and labeling feelings, generalization activities and techniques that support the application of new skills during the school day, and parent materials that aim to extend learning to the home environment. Further information about PATHS can be found at www.pathseducation.co.uk/. The PATHS materials used in the current study were subjected to a process of cultural adaptation by Barnardo’s (the children’s charity who own the UK license to distribute PATHS) in order to ‘Anglicize’ them. These primarily surface level changes (e.g., modified vocabulary, photographs and names, changes to cultural references) did not substantively change the structure or delivery model of PATHS.

Several randomized trials have found small-to-moderate but practically meaningful effects of PATHS on a range of outcomes, including children’s social and emotional skills (Domitrovich et al. 2007), their mental health (Crean and Johnson 2013), and academic attainment (Schonfeld et al. 2015). However, reflecting the trends noted above, some existing studies of PATHS only provide descriptive implementation data (e.g., Domitrovich et al. 2007). Those PATHS studies where implementation-outcomes analyses have been conducted have, in many ways, led the SEL field in terms of our understanding of implementation. However, findings across such studies have been somewhat inconsistent. Some have found little or no connection between levels of PATHS implementation and outcomes (e.g., Berry et al. 2016; Social and Character Development Research Consortium 2010), while others have found significant associations (e.g., Faria et al. 2013; Schonfeld et al. 2015). These studies all maintained a relatively narrow focus, measuring only one or two implementation dimensions, and none assessed reach or participant responsiveness. Finally, with a few exceptions (Conduct Problems Prevention Research Group 1999; Kam et al. 2003; Social and Character Development Research Consortium 2010), existing studies of PATHS implementation have relied exclusively on teachers’ self-reports to generate implementation data. While convenient, this method is, as noted above, limited by the substantial positive bias shown by teachers in their self-ratings and its generally weak relation with more rigorous independent observer ratings (Hansen et al. 2014).

## The Current Study

In 2012, the authors were commissioned to conduct a 2-year cluster-randomized trial of the PATHS curriculum in primary schools in Greater Manchester, England (ISRCTN85087674). Having already published reports on the outcomes of this trial (which were somewhat varied, with evidence of intervention effects on social-emotional skills, mixed findings in relation to mental health, and null results in relation to academic progress—see Humphrey et al. 2015, 2016a), we turn our attention here to assessing the role of variability in PATHS implementation as a moderator of students’ social and emotional skills and mental health outcomes in the first year of the study. In doing so, we sought to advance the current literature in terms of adopting a more wide-ranging approach to the assessment of implementation, increased objectivity and rigor afforded by the use of independent observational data, and the application of a theoretical framework for implementation that posits quality and fidelity as distinct dimensions, enabling us to concurrently assess the relative importance of *what* is delivered and *how well* in determining intervention outcomes (Berkel et al. 2011). These contributions are a direct response to calls for research of this kind in implementation science (e.g., Durlak 2010; Hansen 2014).

## Method

### Design

**Fig. 1:**
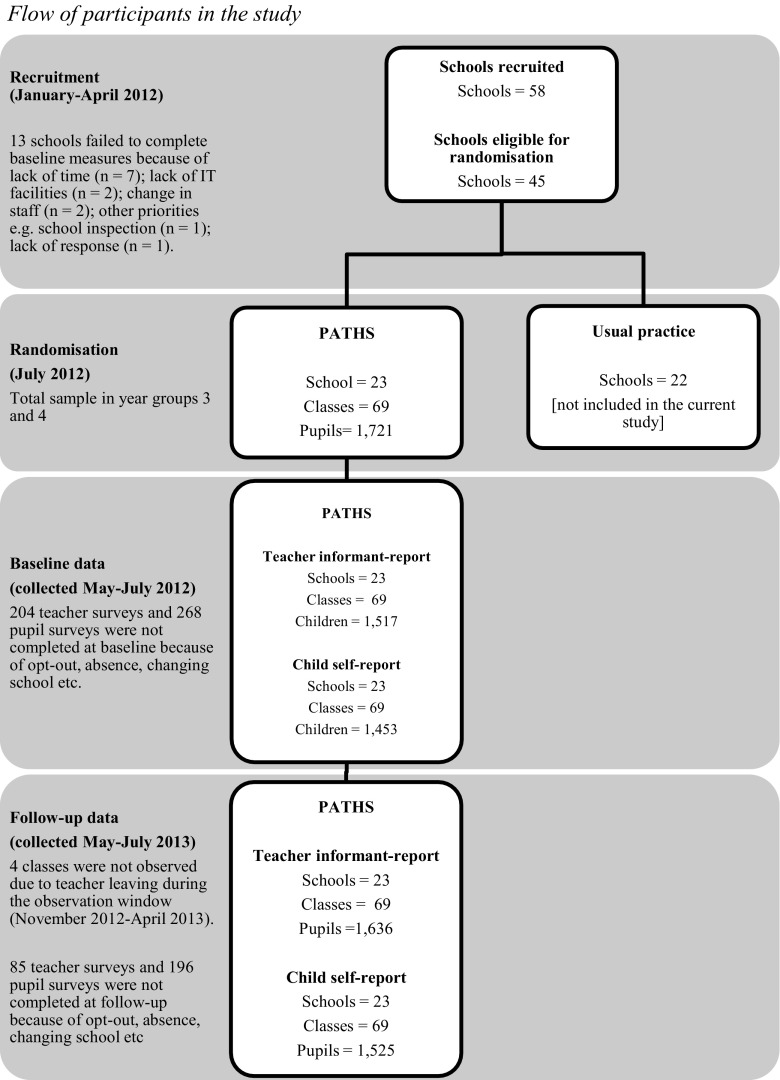
Flow of participants in the study

A longitudinal, natural variation design was utilized. A multi-method approach was taken, with structured observations and surveys as the primary vehicles for data generation in relation to implementation and intervention outcomes respectively. Outcomes were assessed at baseline (summer term 2012) and 12-month follow-up (summer term 2013), with structured observations of implementation taking place in between (specifically, autumn term 2012 and winter term 2013). See Fig. 1 for flow of participants in the study.

### Participants

#### Schools and Teachers

Data were drawn from 23 primary schools implementing PATHS across the Greater Manchester region in the northwest of England. Participating schools were representative of norms in England in respect of size, attendance, attainment, ethnicity, and the proportion of children identified as having special educational needs but had moderately higher proportions of children eligible for free school meals (FSM) and speaking English as an additional language (EAL) than national averages (Department for Education 2012).

Implementation data from 69 Year 3 and 4 teachers/classrooms in the first year of the aforementioned trial were collected. Classes contained an average of 25 students. Teachers of these students averaged 8 years of experience in the classroom, were predominantly female (82.5%), educated to postgraduate level (61.5%), and reported having 2–5 years of experience implementing other SEL programs prior to becoming involved in the current study (40.7%).

#### Students

Outcome data were generated for *N* = 1721 (839 male, 882 female) students, whose average age was 7 years, 7 months (range 6 years, 7 months to 8 years, 10 months) at baseline. Their demographic characteristics were consistent with national norms, albeit with the same exceptions noted above regarding the school characteristics (Department for Education 2012). The proportion of children scoring in the borderline/abnormal ranges for mental health difficulties broadly mirrored national norms for children aged 5–10 (www.sdqinfo.com).

#### Ethics

Participation in the study required consent from schools’ head teachers. Child assent and parental opt-out consent were also sought. Ethical approval from the University of Manchester Research Ethics Committee was sought and received (Ref 11470). In total, 88 parents (5.1% of the sample utilized in the current study) exercised their right to opt their children out of the research, and no children declined assent or exercised their right to withdraw.

## Measures

### Implementation

PATHS lessons were observed by three research assistants, who were each qualified and experienced teachers trained to Masters level in psychology or education. A structured observation schedule was developed by the authors for the study, drawing on the aforementioned theoretical framework for implementation (Berkel et al. 2011), existing rubrics utilized in previous studies of PATHS (e.g., Kam et al. 2003), advice from the program developer and colleagues at Pennsylvania State University, and the extant literature on assessment of implementation (e.g., Domitrovich et al. 2010). Two factual indicators—one each for dosage and reach—were generated and supplemented by ten observer-rated indicators designed to assess fidelity, quality, and participant responsiveness (see Table 1). The data generated was demonstrative of a general trend in which PATHS was delivered between once and twice a week, with most children in a given class present, teachers adhering to most procedural elements outlined in lesson materials, delivering them well, and with children responding appropriately.

**Table 1:** Descriptive statistics and exploratory factor analysis of PATHS implementation indicators

				Factor	
	Scoring	Mean (SD)	Initial designation	Quality and responsiveness	Procedural fidelity
Projected dosage (% lessons complete by end of the school year) based on progress against the implementation schedule?	0–100	65.18 (17.43)	Dosage	–	–
Proportion of the class present during the lesson?	0–100	91.86 (1.10)	Reach	–	–
To what extent does the teacher cover the general and specific objectives of the lesson?	0–10	8.74 (1.46)	Fidelity	0.57	0.48
To what extent does the teacher follow the structure and sequence of activities outlined in the lesson guidance?	0–10	8.04 (2.33)	Fidelity	0.09	0.96
How closely does the teacher adhere to the guidance when teaching the core activities of the lesson?	0–10	7.37 (2.22)	Fidelity	0.08	0.87
How well prepared is the teacher for the lesson?	0–10	8.68 (1.29)	Quality	0.74	0.30
Rate the teacher’s interest and enthusiasm in his/her delivery of the lesson.	0–10	8.99 (1.11)	Quality	0.72	0.18
How clearly does the teacher explain key concepts and activities in the lesson?	0–10	8.41 (1.28)	Quality	0.81	0.21
How well does the teacher respond to pupil queries/ meet the needs of all of the class if it is required?	0–10	8.47 (1.35)	Quality	0.82	0.04
Rate the extent to which children in the class actively participate in the lesson activities.	0–10	7.46 (1.45)	Responsiveness	0.77	−0.01
Rate the level of sustained interest and attentiveness among children in the class during the lesson.	0–10	6.82 (1.85)	Responsiveness	0.84	−0.01
Rate the extent to which the learning objectives have been met.	0–10	7.56 (1.51)	Responsiveness	0.87	0.21

The schedule and an accompanying explanatory rubric^1^ were explained in detail to the research assistants ahead of piloting and refinement using video footage of PATHS lessons being implemented in English schools in a previous trial (Berry et al. 2016). In this initial formative stage, which lasted several days, the emphasis was on developing a shared understanding of the various implementation indicators and their application in the context of a PATHS lesson. Additional video footage of PATHS lessons was then used in order to generate interrater reliability data for each indicator. Given the multiple raters and the ordinal response format of the coding schedule, the intraclass correlation coefficient (ICC) was used. ICC values can range between −1 and 1, with higher scores being indicative of limited variation between raters. The overall ICC was determined to be 0.91, considered to be “excellent” (Hallgren 2012).

During the live trial observations, each teacher was observed implementing a single PATHS lesson at a mutually agreeable date and time. The third author moderated a randomly selected 10% of these observations in order to guard against drift over time. In order to streamline analyses and thus reduce the likelihood of “model overfitting” (Myung 2000), avoid collinearity, and establish clear differentiation between implementation constructs, the observer-rated implementation data were subjected to exploratory factor analysis (EFA) in SPSS using the Principal Axis Factoring extraction method (common factor analysis) with Varimax rotation (oblique rotation method).^2^ The EFA identified two distinct factors, accounting for 69.4% of the explained common variance in the data, corresponding to *procedural fidelity* (α = 0.93) and *quality and responsiveness* (α = 0.93), respectively (see Table 1). Bivariate correlation analyses demonstrated that the two identified factors were clearly distinct from one another (*r* = .02, *p* = .85) and from the dosage and reach indicators (quality-dosage, *r* = −.02, *p* = .79; fidelity-dosage, *r* = −.04, *p* = 0.64; quality-reach, *r* = .08, *p* = .38; fidelity-reach, *r* = .16, *p* = .07), which in turn shared a weak, albeit statistically significant association with each other (*r* = .20, *p* = .02).

To preserve the loading values of each item on a given factor, factor scores were generated using the least squares regression approach (DiStefano et al. 2009). These factor scores, which are standardized to a mean of zero, were subsequently used as explanatory variables for procedural fidelity and quality and responsiveness in the main analysis. To facilitate interpretation within and across models, dosage and reach data were also standardized (e.g., converted to z-scores) (Low et al. 2016).

### Child Self-Report Version of the SSIS

The 46-item Social Skills Improvement System (SSIS) provides a broadband index of children’s social-emotional skills (Gresham & Elliot, 2008). The respondent reads a statement (e.g., “I make friends easily”) and indicates their level of agreement on a four-point scale (never, sometimes, often, always). The instrument is psychometrically sound, with good reliability (internal α up to 0.95; test-retest *r* up to 0.92) and strong validity (factorial: established through confirmatory factor analysis, CFA; convergent: correlates with a range of similar instruments; discriminative: discriminates between clinical and non-clinical samples) (Humphrey et al. 2011). Internal consistency of the SSIS total social skills scale used in the current study was α = 0.92.

Teacher informant-report version of the Social and Emotional Competence Change Index (SECCI). The five-item SECCI was derived from the PATHS program evaluation tools (EPISCenter 2014). Respondents indicate the degree of change they have observed in a child (e.g., “The student’s ability to stop and calm down e.g., when angry, excited or upset”) over a specified period of time using a five-point scale (much worse, a little worse, no change, a little improved, much improved). Internal consistency of this instrument in the current study was α = 0.92.

### Teacher Informant-Report Version of the SDQ

The 25-item Strengths and Difficulties Questionnaire (SDQ) provides a measure of children’s internalizing symptoms, externalizing problems, and pro-social behavior.^3^ Respondents read a statement (e.g., “[This child] often lies or cheats”) and indicate their level of agreement on a three-point scale (not true, somewhat true, certainly true). The SDQ has robust psychometric properties, with evidence of both reliability (internal α up to 0.87; test-retest *r* up to 0.8) and validity (factorial: established through CFA; convergent: correlates with a range of similar instruments; predictive: strongly predictive of independently diagnosed psychiatric disorders) (Goodman et al. 2010; Goodman 2001). Internal consistency in the current study was α = 0.87 for internalizing symptoms, α = 0.90 for externalizing problems, and α = 0.86 for pro-social behavior.

## Statistical Analysis

Outcome data were standardized (e.g., converted to z-scores) prior to analysis. In addition to mean-centring the data, this procedure also facilitates interpretation and produces standardized regression coefficients that are comparable to an effect size that accounts for other variables in the model, thereby increasing precision and rigor (Bierman et al. 2014). In view of the hierarchical and clustered nature of the dataset, we used hierarchical linear modeling in MLWin 2.32. Each model was fitted with two levels (classroom, child), with score at follow-up as the response variable. At the class level, procedural fidelity, quality and responsiveness, dosage, and reach were entered as explanatory variables. Given that there are no universally agreed thresholds of implementation ratings for PATHS (or indeed any school-based intervention; any that have been imposed in studies to date are arguably arbitrary; Berry et al. 2016), we used the observational data to classify each class/teacher as either “low,” “moderate,” or “high” for each aspect of implementation using a distributional cut-point method (low, <−1 SD; moderate, −1 to +1 SD; and high, >+1 SD; in subsequent dummy coding, low implementation was the designated reference group). Importantly, these designations were statistical rather than qualitative (that is, they are based on relative position in the distribution as opposed to being based on arbitrarily imposed thresholds of what “good” implementation might look like; Durlak and DuPre 2008). An exception to this was *reach*: this was coded as high (100%), moderate (90–99%), or low (89% or less) according to the proportion of students present during the PATHS lesson being observed. Descriptive statistics pertaining to these implementation subgroups are available in Table 2.

**Table 2:** Descriptive statistics (*n*, means, and SDs) for implementation subgroups

	Low	Moderate	High
Dosage	11/40.0% (6.76)	48/66.46% (9.92)	7/96.26 (12.88)
Reach	11/72.72% (1.49)	26/90.39% (0.14)	29/100.0% (0.0)
Quality and responsiveness	11/6.29 (0.46)	44/8.21 (0.77)	11/9.57 (0.18)
Procedural fidelity	6/2.00 (2.07)	53/8.02 (1.03)	7/10.0 (0.0)

Given their established associations with social-emotional skills and mental health outcomes (e.g., Green et al. 2005), gender and FSM eligibility were entered as covariates alongside baseline outcome scores at the child level. Guidance on power and sample size for hierarchical linear modeling suggested that the level-two (classroom) sample should be the principal focus given that the primary aim of our analysis was to test the effects of variables at this level (Snijders 2005); here, the level-two sample was deemed sufficiently large to support the explanatory variables noted above (Green 1991; Snijders and Bosker 2012).

Implementation data was missing at the classroom level in 6% of cases, where teachers left the school during the observation window. At the child level, outcome data was missing at either baseline or follow-up for between 13% (SECCI) and 30% (SSIS) of the sample due to student absence or them having left a given school. Missing value analysis showed the data was not missing completely at random (MCAR) but was instead conditional on other variables (e.g., pupil outcome data was more likely to be missing at follow-up). Therefore, the data was considered missing at random (MAR) (Heijtan and Basu 1996). Accordingly, multiple imputation procedures were carried out in REALCOM-Impute, using the MAR assumption (Carpenter et al. 2011). This enabled us to include both partially and completely observed cases of all 69 teachers/classes and 1721 students in the analysis, thereby reducing the bias associated with attrition. Gender and the constant were entered as auxiliary variables. REALCOM-Impute default settings of 1000 iterations and a burn-in of 100, refresh of 10, were used, following guidance for multi-level imputation with mixed response types (Carpenter et al. 2011).

## Results

Descriptive statistics are presented in Tables 1 (overall implementation data and factor loadings), 2 (implementation data by subgroup), and 3 (outcome data). Inferential statistics are presented in Table 4. In the interests of brevity, only the multiply imputed analyses are shown. Complete case analyses were also performed, but there were no substantive differences in findings. In all cases, inclusion of the explanatory implementation variables significantly improved model fit when compared to “unconditional” models (chi-squared tests of the change in −2*Log-likelihood values were all significant at *p* < .001). The ICC for the models presented in Table 3 ranged from 0.02 (SSIS) to 0.35 (SECCI).

**Table 3:** Descriptive statistics (means and SDs) for teacher (SECCI) and child ratings of social and emotional skills (SSIS) and teacher ratings of pro-social behavior, internalizing symptoms, and externalizing problems (SDQ)

			Scoring	Baseline	Follow-up
SECCI			−2 to +2	–	0.68 (0.63)
SDQ					
	Internalizing symptoms		0–20	2.57 (3.06)	2.51 (2.97)
	Externalizing problems		0–20	4.09 (4.42)	3.72 (4.03)
	Pro-social behavior		0–10	7.80 (2.43)	7.69 (2.34)
SSIS			0–138	106.58 (19.59)	104.33 (19.75)

*SECCI* Social and Emotional Competence Change Index, *SDQ* Strengths and Difficulties Questionnaire, *SSIS* Social Skills Improvement System

**Table 4:** Hierarchical linear models of the associations between levels of implementation and intervention outcomes in the PATHS curriculum

	SECCI			SDQ internalizing			SDQ externalizing			SDQ pro-social			SSIS total		
	*β*_0__*ij*_ = 0.18 (0.37)			*β*_0__*ij*_ = 0.20 (0.30)			*β*_0__*ij*_ = 0.09 (0.19)			*β*_0__*ij*_ = 0.12 (0.29)			*β*_0__*ij*_ = 0.18 (0.17)		
	Co-efficient β	SE	*p*	Co-efficient β	SE	*p*	Co-efficient β	SE	*p*	Co-efficient β	SE	*p*	Co-efficient β	SE	*p*
Class	0.35	0.07	<.01	0.20	0.04	<.01	0.07	0.02	<.01	0.18	0.04	<.01	0.02	0.01	.02
Dosage (compared to low)	−0.06 (if mod) −0.32 (if high)	0.27 0.35	.41 .18	−0.01 (if mod) 0.10 (if high)	0.19 0.26	.48 .35	−0.05 (if mod) 0.02 (if high)	0.13 0.17	.35 .45	0.02 (if mod) −0.52 (if high)	0.18 0.26	.46 .02	−0.25 (if mod) −0.28 (if high)	0.11 0.14	.01 .03
Reach (compared to low)	0.11 (if mod) 0.04 (if high)	0.25 0.26	.33 .44	−0.14 (if mod) −0.22 (if high)	0.20 0.19	.24 .13	0.08 (if mod) 0.12 (if high)	0.13 0.13	.27 .18	−0.20 (if mod) −0.23 (if high)	0.18 0.18	.14 .10	−0.02 (if mod) 0.02 (if high)	0.10 0.10	.42 .42
Quality and responsiveness (compared to low)	−0.05 (if mod) 0.22 (if high)	0.24 0.32	.42 .25	−0.09 (if mod) −0.14 (if high)	0.18 0.23	.31 .27	−0.14 (if mod) −0.26 (if high)	0.12 0.15	.11 .04	0.09 (if mod) 0.14 (if high)	0.17 0.23	.30 .27	0.06 (if mod) −0.13 (if high)	0.10 0.12	.28 .14
Procedural fidelity (compared to low)	0.25 (if mod) 0.14 (if high)	0.31 0.37	.21 .35	−0.04 (if mod) 0.34 (if high)	0.23 0.28	.43 .12	0.07 (if mod) 0.18 (if high)	0.14 0.19	.31 .17	−0.13 (if mod) −0.19 (if high)	0.22 0.27	.28 .24	−0.11 (if mod) 0.11 (if high)	0.12 0.15	.18 .23
Pupil	0.61	0.02	<.01	0.65	0.02	<.01	0.42	0.02	<.01	0.56	0.02	<01	0.74	0.03	<.01
Gender (if female)	−0.01	0.04	.38	0.03	0.04	.23	−0.16	0.04	<.01	0.36	0.04	<.001	0.27	0.05	<.01
FSM (if eligible)	−0.04	0.05	.16	0.10	0.05	.02	0.12	0.04	.01	−0.17	0.05	.01	−0.13	0.06	.01
Baseline score	–	–	–	0.38	0.02	<.01	0.70	0.02	<.01	0.40	0.02	<.01	0.44	0.03	<.01

*SECCI* Social and Emotional Competence Change Index, *SDQ* Strengths and Difficulties Questionnaire, *SSIS* Social Skills Improvement System

### Quality and Responsiveness

Compared to low levels, high levels of implementation quality and participant responsiveness were associated with significantly lower ratings of students’ externalizing problems at 12-month follow-up (β = −0.26, *p* = .04). This effect was mirrored in a marginal, non-significant trend relating to moderate implementation quality and participant responsiveness for the same outcome variable (β = −0.14, *p* = .11). Levels of implementation quality and responsiveness were not significantly associated with any other intervention outcome (all *p* > .05).

### Procedural Fidelity

Levels of procedural fidelity were not significantly associated with any intervention outcome (all *p* > .05).

### Dosage

Contrary to expectations, high levels of dosage (compared to low) were associated with significantly lower ratings of students’ pro-social behavior at 12-month follow-up (β = −0.52, *p* = .02). Similarly, both moderate (β = −0.25, *p* = .01) and high (β = −0.28, *p* = .03), compared to low levels of dosage, were associated with significantly lower ratings of students’ social-emotional skills. Levels of dosage were not significantly associated with any other intervention outcome (all *p* > .05).

### Reach

Levels of reach were not significantly associated with any intervention outcome (all *p* > .05).

## Discussion

The principal aim of the current study was to assess the relationship between implementation and intervention outcomes in the PATHS curriculum. In doing so, we sought to offer distinct contributions to the field by adopting a more wide-ranging approach to the assessment of implementation than has previously been evident, through the increased objectivity and rigor afforded by the use of independent observational data, and via the application of a theoretical framework for implementation that posits quality and fidelity as distinct dimensions, enabling us to concurrently assess the relative importance of *what* is delivered and *how well* in determining intervention outcomes (Berkel et al. 2011). Our analysis of observational implementation data revealed distinct dimensions of implementation *quality and responsiveness* and *procedural fidelity*. Implementation-outcomes analyses demonstrated that high (and, marginally, moderate) levels of implementation quality and responsiveness were associated with significantly lower ratings of students’ externalizing problems. Contrary to expectations, high (and, for students’ social-emotional skills, moderate) levels of dosage were associated with significantly lower ratings of students’ pro-social behavior and social-emotional skills. No associations were found between variability in either procedural fidelity or reach and intervention outcomes.

The factor analytic model of our observational data offers clear empirical support for the integrated model of implementation that posits fidelity and quality as distinct dimensions (Berkel et al. 2011). The fact that the observational indicators relating to quality and those relating to participant responsiveness loaded strongly together in our EFA^4^ is also consistent with the integrated model, in which the former is seen as a foundation for the latter. It is here where parallels with the literature on therapeutic alliances may be drawn; in this body of work, the relational and interactional bond between therapist and client is articulated in terms the competence and skills of the former and the engagement and active participation of the latter. Interestingly, the quality of this alliance has been shown to be a reliable predictor of positive intervention outcomes regardless of the therapeutic model used (Ardito and Rabellino 2011). Thus, as in the current study, *quality matters*.

Our implementation-outcomes analyses challenge the predominance of fidelity and dosage in the study of school-based interventions. Elsewhere, we have argued against the “zero sum game” view of implementation (e.g., fidelity is all that matters, and therefore attention to matters beyond fidelity is not worthwhile; Elliott and Mihalic 2004) on conceptual and theoretical bases (Lendrum et al. 2016). Here, we extend our position by demonstrating empirically that variability in procedural fidelity appears to be unrelated to intervention outcomes in the PATHS curriculum. Our findings align with those of Berry et al. (2016) and the Social and Character Development Research Consortium (2010). These authors also found no association between fidelity and outcomes in their recent PATHS trials. However, it is important to note that their analyses did not take account of the critical distinction between fidelity and quality made in the current study.

Our findings contrast with those of Faria et al. (2013) and Schonfeld et al. (2015), both of whom found a significant, *positive* association between PATHS dosage and outcomes. The dosage levels reported in the current study (see Table 1) are comparable with those of Faria et al. (2013),^5^ so the apparent *negative* effect seen here is presumably not because of a failure to achieve a “minimum effective dose” (Liu 2010). Instead, we speculate that methodological and other differences between our studies may account for the apparent incongruence. For example, Schonfeld et al. (2015) used different methods to assess implementation (teacher self-report), covered a longer period of implementation (up to 4 years), and assessed different outcomes (academic attainment) than the current study.

Such differences aside, the question still remains as to why higher levels of dosage were found to be associated with significantly worse intervention outcomes. One possible reason is that this high dosage was at the expense of quality. Put another way, some teachers may have engaged in a “race to the finish line,” implementing PATHS *quickly* rather than implementing it *well*. An alternative explanation is that the teachers who implemented PATHS more frequently did so because they had a lower functioning class. In support of this hypothesis, exploration of the study data indicated that children in the moderate and high dosage classrooms demonstrated marginally higher internalizing symptoms, externalizing problems, and lower pro-social behavior at baseline. Finally, it may simply be the case that more frequent delivery of PATHS lessons meant that other efficacious activities (including, potentially, targeted interventions) were displaced.

Conversely, we found that higher implementation quality and participant responsiveness was associated with lower ratings of students’ externalizing problems at 12-month follow-up. These analyses support Durlak’s (2015) claim that “in some circumstances, quality of delivery… may be more strongly related than other implementation components to some program benefits” (p.1126) and add to a small but growing evidence base on the importance of this dimension of implementation as a moderator of intervention outcomes. In particular, our findings support those of Pettigrew et al. (2015), whose implementation-outcomes analyses of the *keepin’ it REAL* program revealed that implementation quality and participant responsiveness were more reliable predictors of intervention outcomes than fidelity. This emergent pattern of findings suggests that a broadening of focus to incorporate quality and responsiveness is perhaps warranted in implementation support processes (e.g., initial training, on-going technical support and assistance). This may, however, prove to be challenging for manualized interventions that perhaps lend themselves to a more procedural emphasis.

The current study is not without limitations. Chief among these was the fact that we were only able to observe each teacher/classroom once, thereby providing only a “snapshot” of implementation. The general recommendation is to capture implementation over multiple occasions to improve reliability and such that temporal patterns can be identified and taken into account in analyses (Humphrey et al. 2016b). As a counterpoint, however, we note the fact that some major observational studies of temporal patterns in implementation have actually evidenced high levels of stability in key dimensions (e.g., Hansen et al. 2013). Of particular relevance is Domitrovich et al.’s (2010) study of PATHS, which found no significant changes in fidelity, dosage, or participant responsiveness when growth models were applied to implementation data collected monthly over the course of a school year. Similar temporal stability (in implementation quality) was found in the FAST Track trial of PATHS (CPPRG 1999). Moreover, multiple observations in the current study were simply not possible due to resource and data burden considerations. We do note though that, as observations were scheduled with teachers, they might have differentially prepared for these lessons. This is, however, almost impossible to avoid, given the ethical and practical considerations inherent in observational studies of implementation in schools.

A second limitation is that, despite adopting a more wide-ranging approach to the assessment of implementation than had previously been evident, the current study was not completely comprehensive. It is difficult, if not impossible, to study all implementation components simultaneously (Durlak 2016). Specifically, we were not able to include data on program differentiation or adaptations in the analyses reported here. In terms of the former, establishing the distinctiveness of a given intervention from existing classroom practice is crucial in terms of determining its “achieved relative strength” (Nelson et al. 2012). In relation to the latter, assessment needs to take into account the reasons for adaptation (e.g., logistical, philosophical), their timing (e.g., pro-active, reactive), and valence (e.g., positive, negative, neutral) (Moore et al. 2013). These two dimensions have proven particularly elusive in the broader field (Humphrey et al. 2016b). However, recent work by Hansen et al. (2013) suggests that reliable and valid assessment is possible, albeit time consuming and costly.

Finally, we should also note alternative explanations for the lack of positive associations between procedural fidelity, dosage, and the outcomes modeled herein. It is possible, for example, that once minimum effective levels of these dimensions of implementation are reached, little or no “added value” is gained from higher levels. While somewhat plausible, this explanation does not align well with findings of other studies (e.g., the aforementioned study by Schonfeld et al. (2015) found that the probability of achieving academic proficiency status in reading increased 1.37 times for each additional lesson taught), and is also discordant with the developers’ recommendations, particularly in relation to dosage. Another explanation is that there was not enough variability in our dataset, particularly for procedural fidelity, to detect its effects. However, scrutiny of the descriptive data for the implementation indicators (Table 1) does not support this interpretation, as the two procedural fidelity indicators actually yielded *higher* standard deviations (indicative of greater variability) than the quality and responsiveness indicators.

## Conclusion

The current study adds to the growing body of literature exploring the relationship between implementation and intervention outcomes in school-based SEL. We provide distinct contributions in terms of the adoption of a more wide-ranging approach to the assessment of implementation than has previously been evident, increased objectivity and rigor afforded by the use of independent observational methods, and the application of a theoretical framework for implementation that posits quality and fidelity as distinct dimensions. Our analyses provide support for the integrated model of implementation and suggest that quality and responsiveness are at least as critical as fidelity and dosage, if not more so, in determining the achievement of expected outcomes. Put another way, the current study reinforces Durlak’s (2010) clarion call for the advancement of implementation science, in which he noted “the importance of doing well in whatever you do” (p.348).

## Footnotes

1 The rubric was designed to orient observers to behaviors indicative of the implementation indicator in question. For example, when rating students’ engagement in core activities, the explanatory notes guided observers to assess the extent to which children in the class actively participated in lesson activities (e.g., joining in role plays and answering questions).

2 In order to meet minimal sample size requirements (e.g., *N* = 10 per item), the EFA was conducted using data from the 127 observations conducted across the 2 years of the trial.

3 Historically, the SDQ has been scored according to a five-factor structure (emotional symptoms, conduct problems, inattention/hyperactivity, peer problems, and pro-social behavior). However, research has indicated that a three-factor structure (internalizing symptoms, externalizing problems, pro-social behavior) offers improved data fit (Goodman, Lamping & Ploubidis, 2010).

4 We note that one item initially designated as belonging to the fidelity dimension also loaded onto this factor; however, there was evidence that of ambiguity, with this item cross-loading onto both factors (loadings of 0.57 and 0.48 respectively).

5 Schonfeld et al. (2015) did not provide descriptive statistics for their implementation data and a direct comparison of overall dosage levels is therefore not possible.
